# Roles of OB-Fold Proteins in Replication Stress

**DOI:** 10.3389/fcell.2020.574466

**Published:** 2020-09-11

**Authors:** Dinh-Duc Nguyen, Eugene Y. Kim, Pau Biak Sang, Weihang Chai

**Affiliations:** Department of Cancer Biology, Cardinal Bernardin Cancer Center, Loyola University Chicago Stritch School of Medicine, Maywood, IL, United States

**Keywords:** OB-fold protein, single strand DNA-binding protein, replication fork, replication stress, BRCA2, CST, RPA, genome stability

## Abstract

Accurate DNA replication is essential for maintaining genome stability. However, this stability becomes vulnerable when replication fork progression is stalled or slowed – a condition known as replication stress. Prolonged fork stalling can cause DNA damage, leading to genome instabilities. Thus, cells have developed several pathways and a complex set of proteins to overcome the challenge at stalled replication forks. Oligonucleotide/oligosaccharide binding (OB)-fold containing proteins are a group of proteins that play a crucial role in fork protection and fork restart. These proteins bind to single-stranded DNA with high affinity and prevent premature annealing and unwanted nuclease digestion. Among these OB-fold containing proteins, the best studied in eukaryotic cells are replication protein A (RPA) and breast cancer susceptibility protein 2 (BRCA2). Recently, another RPA-like protein complex CTC1-STN1-TEN1 (CST) complex has been found to counter replication perturbation. In this review, we discuss the latest findings on how these OB-fold containing proteins (RPA, BRCA2, CST) cooperate to safeguard DNA replication and maintain genome stability.

## Introduction

Faithful and accurate duplication of DNA is important for passing genetic material to the subsequent generation. This process is coordinated by a group of events and proteins in the nucleus to safeguard cellular DNA synthesis. Replication stress (RS) is broadly defined as the slowing or stalling of replication fork progression and/or DNA synthesis (Zeman and Cimprich, 2014). RS can be caused by either intrinsic sources arising from cellular metabolism processes or extrinsic sources from environmental exposure. RS threatens genome stability and gives rise to cancer and other pathological diseases (Zeman and Cimprich, 2014).

To prevent genome instability caused by RS, cells activate the ataxia telangiectasia and Rad3-related (ATR)-mediated DNA damage response pathway to sense stalled replication and arrest the cell cycle to rescue replication. ATR is a serine/threonine protein kinase that belongs the phosphatidylinositol 3 (PI3) kinase family. It is activated by replication protein A (RPA) binding to ssDNA formed at stalled forks. Upon activation, ATR phosphorylates a series of downstream effectors including checkpoint kinase 1 (CHK1), and triggers a cascade of signals to promote cell cycle arrest and resolve RS through multiple pathways including fork remodeling, dormant origin firing, template switching and replication repriming (Zou et al., 2006; Zeman and Cimprich, 2014; Blackford and Jackson, 2017; Bhat and Cortez, 2018).

Upon fork stalling, DNA polymerases slow down while helicases continue unwinding DNA. This results in the formation of excessive ssDNA that is unstable and can be easily attacked by endonucleases (Zeman and Cimprich, 2014; Kitao et al., 2018). To avoid damages to the genome, these ssDNA stretches are safeguarded by highly dynamic ssDNA binding proteins. Among them, a group of these proteins contain Oligonucleotide/oligosaccharide binding (OB)-fold domains. These OB-fold ssDNA-binding proteins play three main critical roles: preventing ssDNA from re-annealing, protecting ssDNA from degradation, and providing signals for subsequent cellular pathways to decide which repair pathways should be activated, all of which are critical to the re-initiation of DNA synthesis and preserving genome integrity (Bochkareva et al., 2002; Haring et al., 2008; Chastain et al., 2016; Belanger et al., 2018; Ibler et al., 2019).

**FIGURE 1:**
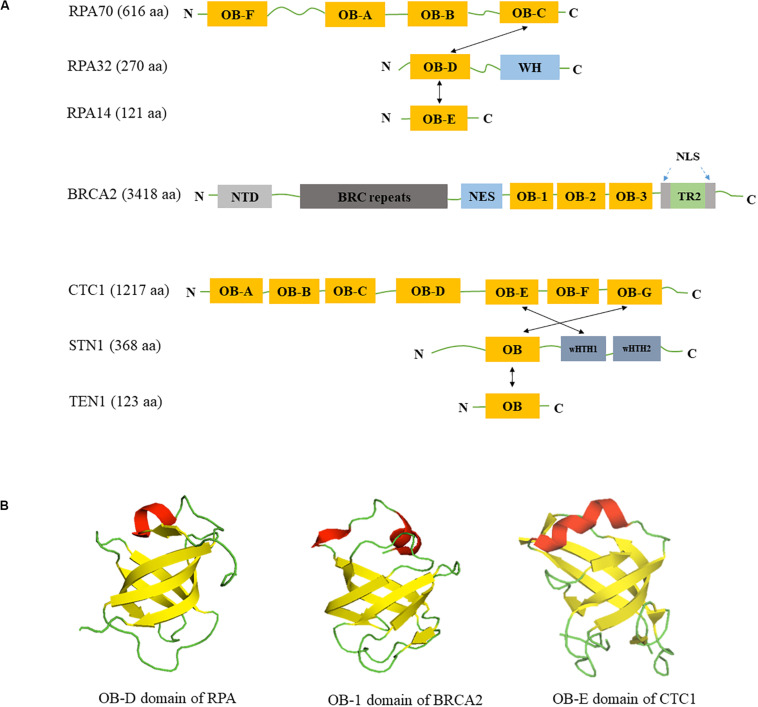
Domain structures of RPA, BRCA2, CST. **(A)** Domain structures of RPA, BRCA2, and CST. OB: OB-fold domain; WH: winged helix domain; NTD: N-terminal domain; BRC repeats: BRCA2 exon 11 encodes eight conserved motifs; NES: nuclear export signal domain; NLS: nuclear localization signals; TR2: the single RAD51-binding domain; wHTH: winged helix-turn-helix domain; Black arrows show the intermolecular interactions between subunits. **(B)** Similarity of OB-fold structures in RPA, BRCA2 and CST. OB folds are β barrels formed by five antiparallel β-sheets. β-strands are colored in yellow, α-helices are colored in red and loops are colored in green. Structures are derived from Protein Data Bank with structure codes 1L1O (RPA), 1IYJ (BRCA2), and 5W2L (CTC1).

The OB-fold family has been well characterized since four proteins that bind either oligonucleotides or oligosaccharides were discovered in 1993 (Murzin, 1993). To date, 1552 proteins containing OB-fold structural domains have been deposited in the Protein Data Bank (updated by April 30th, 2020). Characterization of the first OB structure shows that the OB-fold is formed by at least five β-strands arranged in an anti-parallel manner, shaping into a β-barrel that is captured by an α-helix capping (Murzin, 1993; Figure 1B). Since then, it has been discovered that the OB-fold structure is highly dynamic (Arcus, 2002; Bochkarev and Bochkareva, 2004). Loops linking β-strands can adopt different conformations to open or close the β-barrel (Bycroft et al., 1997). Additionally, the α-helix capping of the barrel can change to an extended loop or a three-bundle helix (Arcus, 2002; Bochkareva et al., 2002). Although it is notorious for the lack of primary sequence conservation, the OB-fold motif supports a similar dynamic binding surface for protein-protein interaction and protein-ssDNA binding (Figure 1B). The dynamic properties of the OB-fold structure allow OB-fold containing proteins to participate in multiple cellular pathways including genome maintenance as mentioned above. Understanding the structures of OB-fold proteins and their functions in RS response may provide a therapeutic approach for cancer and other human diseases caused by defective RS response.

In this review, we summarize and discuss the latest findings on structural properties and functions of three important OB-fold proteins/protein complexes – the well characterized RPA proteins (comprising of RPA70, RPA32, RPA14) and breast cancer susceptibility protein 2 (BRCA2), as well as the new member CST – in countering RS and protecting genome stability. RPA participates in RS response via its binding to ssDNA (Wold, 1997; Zou et al., 2006; Glanzer et al., 2014; Belanger et al., 2018). Structure of the OB-A domain of RPA70 was solved early and has been used for characterizing other OB-fold proteins (Bochkarev et al., 1997), including BRCA2 and CST that have been implicated in RS response (Yang et al., 2002; Bochkarev and Bochkareva, 2004; Sun et al., 2009; Wang and Chai, 2018; Lyu et al., 2019b). Through binding to ssDNA and their protein binding partners at stalled forks via OB-fold domains, these proteins influence the remodeling of stalled forks, modulate the activities of other important proteins at forks, and/or act as signal responders to fork stalling. Lastly, we will discuss their implications in cancer therapeutics.

## RPA

RPA is an essential regulator in the DNA replication process. Its binding to ssDNA not only protects ssDNA from nucleolytic degradation but also forms a platform facilitating the recruitment of many binding partners for diverse functions. Here, we discuss the latest findings on the dynamic binding of RPA to ssDNA and its binding partners in RS response.

### RPA Protein Structure and Its DNA Binding Properties

The canonical RPA complex is a heterotrimer complex containing three subunits: RPA70, RPA32, and RPA14 with molecular mass of 70, 32, and 14 kDa, respectively. RPA70 contains four different OB-fold domains OB-A, OB-B, OB-C, and OB-F (Figure 1A). RPA32 is composed of one OB-fold domain OB-D at its N-terminus and a winged helix (WH) domain at the C-terminus (Figure 1A). The smallest subunit RPA14 contains one OB-fold domain, OB-E (Figure 1A). The three RPA subunits form a trimerization core structure through the interactions between OB-C/OB-D/OB-E domains (Bochkareva et al., 2002; Cai et al., 2007; Deng et al., 2007). The high binding affinity of RPA to ssDNA is mostly mediated by four OB-fold domains OB-A, OB-B, OB-C, and OB-D in RPA70 and RPA32, while OB-F and WH domains are responsible for interacting with its protein binding partners (Kim et al., 1994; Bochkareva et al., 2002; Fanning et al., 2006; Jiang et al., 2006). In addition, the OB-fold domains are connected by mobile loops that make RPA a flexible complex, permitting its six OB-fold domains to adopt multiple conformations (Yates et al., 2018).

Recently, a study using the single-molecular Forster Resonance Energy Transfer (FRET) technique reveals that RPA-DNA binding is highly dynamic and involves at least three distinct binding modes (Wang Q. M. et al., 2019). The three modes are designated as 10, 20, and 30 nt binding modes that help to explain the dynamic binding of RPA to ssDNA that is dependent on RPA concentration and ssDNA length (Wang Q. M. et al., 2019). These findings are consistent with previous studies (Fanning et al., 2006; Chen and Wold, 2014; Bhattacharjee et al., 2017; Pokhrel et al., 2019) and suggest that RPA is capable of adjusting its binding modes within a broad range of concentrations. The dynamic binding of RPA allows it to adopt different conformations on ssDNA or rapidly diffuse along ssDNA to destabilize secondary structures that can cause RS. In addition, RPA-ssDNA binding provides the nucleation sites for RPA displacement by other proteins in homologous recombination (HR). However, exactly how such dynamic binding to ssDNA affects RPA’s biological functions remains to be elucidated.

With a flexible structure and versatile DNA binding modes, RPA actively helps channel different ssDNA intermediates into separate pathways in the cell, including RS response and DSB repair. These multiple binding mechanisms, including ssDNA and its co-factor interactions, provide distinctive functionalities to ensure that appropriate activities are promptly deployed to overcome DNA damage and replication challenges (Table 1; Wyka et al., 2003; Zou et al., 2006; Chen and Wold, 2014; Glanzer et al., 2014; Pokhrel et al., 2019).

**TABLE 1:** The binding partners of OB-fold proteins (RPA, BRCA2, and CST) and their roles in DNA replication process.

	**Binding Partner**	**Function**	**References**
RPA	ATR-ATRIP, RAD52, SNEP6, TOPBP1, ETAA1, MRN	Activate/stimulate the ATR signaling	Robison et al., 2004; Kumagai et al., 2006; Seong et al., 2009; Dou et al., 2010; Lee et al., 2016; Ma et al., 2017; Lyu et al., 2019a
	HDHB	RPA-binding stimulates accumulation of HDHB on chromatin in RS	Guler et al., 2012
	WRN	RPA-binding promotes WRN helicase activity and multiple RPA binding makes WRN a superhelicase on G4 unwinding	Brosh et al., 1999; Lee et al., 2018
	BLM	RPA activates BLM’s bidirectional DNA unwinding	Qin et al., 2020
	SMARCAL1	SMARCAL1 is recruited to replication forks via an interaction with RPA	Bhat et al., 2015
	PrimPol	RPA enhances PrimPol primase/polymerase activity at forks	Guilliam et al., 2017; Martinez-Jimenez et al., 2017
	RNaseH1	RPA colocalizes with R-loops and suppresses R-loop formation	Nguyen et al., 2017
BRCA2	RAD51	BRCA2 replaces RPA-bound ssDNA with RAD51 to form nucleofilaments at replication forks for FP or at DNA breaks for HR-mediated repair	Bork et al., 1996; Bignell et al., 1997; Wong et al., 1997; Esashi et al., 2007
	PALB2	Recruits BRCA2 to the stalled forks or DNA damage sites	Sy et al., 2009; Zhang et al., 2009; Buisson et al., 2014; Hartford et al., 2016
	PLK1, FANCD2, and BOD1L	Assists BRCA2 in RAD51 recruitment	Hussain et al., 2004; Schlacher et al., 2012; Yata et al., 2014; Higgs et al., 2015
CST	Polymerase α primase, TPP1-POT1	CST stimulates the primase activity of POLα and helps in C strand fill-in	Chen et al., 2012; Huang et al., 2012
	MCM Complex	CST disrupts binding of CDT1 to MCM	Wang Y. et al., 2019
	RAD51	CST interacts with RAD51 under RS and stabilizes stalled fork	Chastain et al., 2016
	AND-1	CST interacts with AND-1 and promotes AND-1 and POLα chromatin binding	Wang and Chai, 2018
	Shieldin	CST counteracts DSB end resection, possibly by POLα mediated fill-in	Barazas et al., 2018; Mirman et al., 2018

### RPA-ssDNA Complex as the Signal Responder to Stalled Replication

**FIGURE 2:**
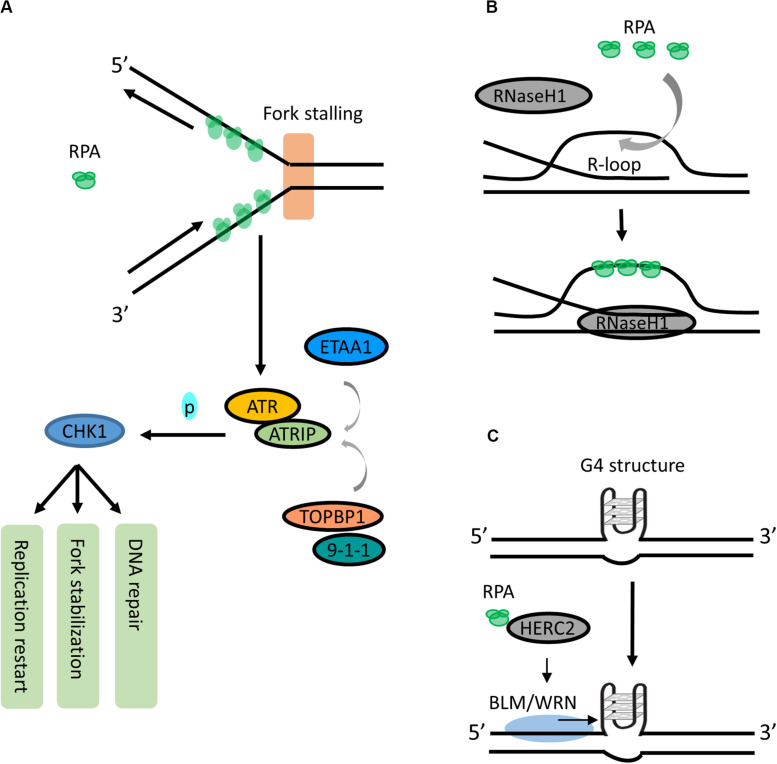
Roles of RPA in RS responses. **(A)** RPA is a signal responder of RS in ATR signaling. During RS response, the RPA-coated ssDNA acts as a key recruitment/activation platform for recruiting ATR-ATRIP to the stalled fork. The kinase activity of ATR-ATRIP is stimulated by TOPBP1:9-1-1 or ETAA1. Activated ATR-ATRIP phosphorylates and activates CHK1, which induces cell cycle arrest to allow DNA repair, fork stabilization or replication start. **(B)** RPA is a sensor for resolving R-loops. RPA may sense the increase of R-loops as an RS signal and promotes RNaseH1 resolving R-loops by recognizing ssDNA within R-loop. **(C)** RPA can unfold the G4 structures. RPA interacts with HERC2 and promotes BLM/WRN helicase to unwind or suppress G4 formation.

This RPA-ssDNA binding is known as the first signal to activate the ATR-signaling pathway during cellular response to RS. At stalled forks or resected double strand breaks (DSBs), RPA-coated ssDNA acts as a key recruitment/activation platform to recruit the ATR-ATRIP (ATR-interacting protein) kinase complex (Zou and Elledge, 2003; Chen and Wold, 2014; Blackford and Jackson, 2017; Figure 2A). Subsequently, the kinase activity of ATR-ATRIP is stimulated by DNA topoisomerase 2-binding protein 1 (TOPBP1) through direct interacting and loading of the 9-1-1 (RAD9-RAD1-HUS1) complex (Kumagai et al., 2006; Mordes et al., 2008; Figure 2A). Activated ATR-ATRIP phosphorylates and induces transcription of numerous downstream targets including tumor suppressor p53 and CHK1, which facilitates cell cycle arrest to stabilize stalled forks, repair DNA damage, restart replication or activate the apoptotic pathway (Smith-Roe et al., 2013; Blackford and Jackson, 2017; Figure 2A).

While the mechanism of ATR:ATRIP-TOPBP1:9-1-1-CHK1 axis has long been well described, new findings have identified a TOPBP1-independent activator of the ATR-ATRIP complex in human cells. Ewing’s tumor-associated antigen 1 (ETAA1) promotes ATR kinase activity via binding to RPA (Bass et al., 2016; Haahr et al., 2016; Lee et al., 2016). It is recruited to stalled forks via two RPA-binding domains and participates in RS response independently from the TOPBP1:9-1-1 complex (Haahr et al., 2016; Lee et al., 2016). Additionally, Lyu et al. (2019a) have shown that binding of ETAA1 to RPA-coated ssDNA directly stimulates its ability to activate ATR-ATRIP, suggesting that RPA-coated ssDNA serves as a direct stimulator in the ETAA1-mediated activation of ATR-ATRIP (Lyu et al., 2019a; Figure 2A). Interestingly, the ATR activation motif in ETAA1 shares similarity to that in TOPBP1, suggesting that TOPBP1 and ETAA1 likely activate ATR using parallel mechanisms (Thada and Cortez, 2019). Together, these findings highlight that RPA-coated ssDNA acts as a critical sensor of RS and actively participates in recruiting different proteins in the ATR signaling pathway.

### RPA in Sensing and Resolving R-loop and G-quadruplex (G4)

R-loop has emerged as a major source of genomic instability. It is a transcription intermediate containing RNA:DNA hybrid resulting from RNA transcript displacing ssDNA. During the S phase, the collision between replication forks and transcription machinery may increase R-loop formation (Gan et al., 2011). Recently, a study shows that RPA is involved in suppressing R-loop formation by directly stimulating the activity of RNaseH1 on R-loops in a concentration-dependent manner (Nguyen et al., 2017). In addition, RPA co-localizes with RNaseH1 at R-loop foci, and this colocalization is required for suppressing R-loop-associated DNA damage (Nguyen et al., 2017). It has been proposed that RPA may sense the increase of R-loops as an RS signal and promotes RNaseH1 resolving R-loops in front of replication forks by recognizing ssDNA within the R-loop structure (Nguyen et al., 2017; Parajuli et al., 2017; Figure 2B). Thus, in addition to sensing ssDNA, RPA is also a sensor of R-loops and a regulator of RNaseH1, extending the versatile role of RPA in suppressing genomic instability.

RPA has also been reported to be able to unfold G4 structures - a stable four-stranded DNA secondary structure formed by the guanine-rich DNA sequences via Hoogsteen base pair bonding. RPA binds and unfolds G4s under physiologically relevant conditions *in vitro* (Salas et al., 2006; Qureshi et al., 2012; Ray et al., 2013). It unwinds G4 from 5′ to 3′, and this unwinding is independent of the number of G4 units (Safa et al., 2016; Lancrey et al., 2018). Interestingly, Wu et al., recently showed that HERC2, a HECT E3 ligase, facilitates BLM (Bloom syndrome helicase) and WRN (Werner syndrome helicase) interaction with RPA and plays a critical function in suppressing G4 formation (Wu et al., 2018; Figure 2C). In addition, binding of RPA to WRN promotes a superhelicase activity of WRN (Lee et al., 2018). Together, these studies suggest an important role of RPA and its interacting partners in resolving G4s in the genome. More investigation is needed to fully understand the binding of RPA to G4s and its binding partners, as well as whether these interactions could navigate G4 unfolding.

### RPA-ssDNA in Promoting DSB Repair Through HR

Another well-described function of RPA is promoting DSB repair during HR. When a replication fork encounters a DNA lesion, DSBs may be generated. Such replication fork-associated DSBs can be repaired by HR. During the early stage of HR, DSB ends are processed by MRN [meiotic recombination 11 (MRE11), RAD50, and NBS1], which produces a 3′ ssDNA. This ssDNA is quickly bound by RPA through the interaction between RPA and MRE11. In order for this interaction to occur, RPA32 phosphorylation, which prevents interaction between RPA and MRN, is removed, thus allowing the OB-F domain of RPA70 to bind to an acidic α-helix peptide in MRE11 (Oakley et al., 2009). RPA loading onto the 3′ ssDNA prevents secondary structure formation and protects ssDNA from degradation.

RPA binding at the resected end serves as an important intermediate for the DNA recombinase RAD51 to form the nucleoprotein filament (Deng et al., 2014; Ruff et al., 2016), which stimulates the HR process with the assistance from other pro-recombinogenic mediators such as RAD52 and BRCA2 (Seong et al., 2009; Daley et al., 2013). Before RAD51 can replace RPA, SENP6, a SUMO-specific protease, is separated from RPA70 after DNA damage, allowing for RPA70 sumoylation. RPA70 is then modified by small ubiquitin-like modifier (SUMO) 2/3, and this modification also promotes RAD51 recruitment to the DNA damage foci during HR (Dou et al., 2010).

The single molecule imaging technology has revealed that human RAD52 binds very tightly to RPA-coated ssDNA (Ma et al., 2017). This binding is restricted by RAD51. When RAD51 is dissociated from the ssDNA, additional RAD52 can bind to the RPA-ssDNA complex (Ma et al., 2017). These results suggest a new insight into the behavior and dynamics of ssDNA-RPA/RAD52/RAD51 interaction. However, the biological relevance of these RPA-RAD52 clusters remains to be determined.

### RPA in Replication Fork Remodeling/Reversal

**FIGURE 3:**
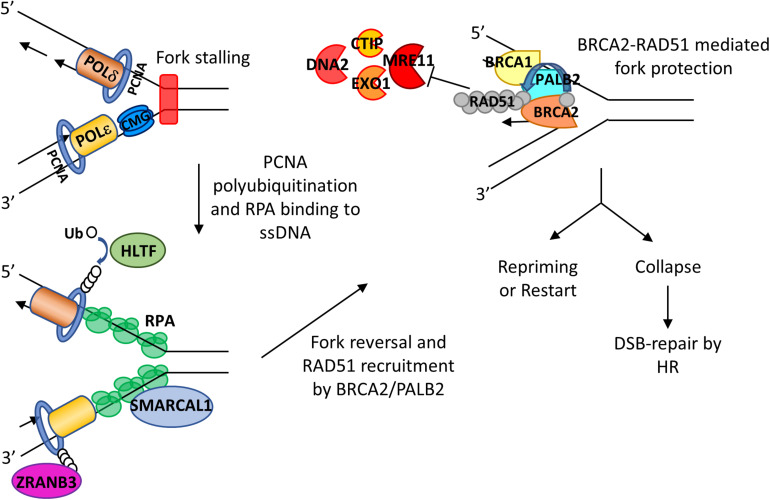
Roles of RPA and BRCA2 in fork reversal and fork protection. RS leads to fork slowing and fork reversal by SNF2 family chromatin remodelers SMARCAL1, ZRANB3, and HLTF. HLTF polyubiquitinates PCNA and thus leads to the recruitment of ZRANB3. SMARCAL1 directly interacts with RPA on the leading strand, and RPA controls the fork remodeling activity of SMACARL1. After fork reversal and RPA phosphorylation, PALB2 binds to RPA and recruits BRCA2. BRCA2 recruits RAD51 with assistance of PLK1 and mediates replacement of RPA with RAD51, leading to fork protection from nucleases such as MRE11, EXO1, CTIP, and DNA2. The fork can then be reprimed or restarted. When undergoing sustained RS, stalled forks may collapse, leading to DSBs that can be subsequently repaired by HR.

When replication forks encounter DNA lesions, fork remodeling/reversal is a key protective mechanism that allows forks to reverse their course without chromosomal breakage (Neelsen and Lopes, 2015). The current model of stalled forks suggests that there are at least two steps involved. First there is a fork reversal, which is the remodeling of forks into a four-way junction, and then protection of the nascent strand through a tightly controlled resection that allows for fork restart (Berti and Vindigni, 2016). The proteins involved in fork reversal include RAD51 (Zellweger et al., 2015; Kolinjivadi et al., 2017; Mijic et al., 2017) and ATPase-dependent DNA translocases of the SWI2/SNF2 family of chromatin remodelers such as SMARCAL1 (Yusufzai and Kadonaga, 2008; Betous et al., 2012, 2013; Ciccia et al., 2012), ZRANB3 (Yusufzai and Kadonaga, 2010; Ciccia et al., 2012; Yuan et al., 2012), HLTF (Blastyak et al., 2010; Kile et al., 2015), and RAD54 (Bugreev et al., 2011). SMARCAL-1 (SWI/SNF-related, matrix-associated, actin-dependent regulator of chromatin, subfamily A-like 1) is a fork-remodeling enzyme. Its fork remodeling activity is controlled by RPA (Bhat et al., 2015). RPA binds to ssDNA at the fork junction, creating an optimal DNA-protein substrate for SMARCAL1 and directing fork regression (Bhat et al., 2015; Figure 3). Interestingly, while RPA binding to ssDNA formed at the leading strand stimulates SMARCAL1-mediated fork remodeling activity, RPA binding at the lagging strand inhibits SMARCAL1 activity (Bhat et al., 2015). The underlying mechanism for such discrepancy is unclear, and whether RPA influences the activities of other fork remodelers remain to be investigated.

### RPA in Regulating Activities of Other Polymerases/Helicases in Response to RS

RPA can interact with polymerases and helicases and regulates the activities of these enzymes. PrimPol (DNA-directed primase/polymerase protein) is a translesion synthesis polymerase containing both the primase and the polymerase activities. When replication is stalled by DNA lesion, PrimPol can be recruited to the stalled site and initiate DNA replication past the site of the lesion. Cells depleted of PrimPol display an increase of spontaneous DNA damage and are defective in restarting stalled replication forks (Wan et al., 2013). Thus, it is believed to be an important player in bypassing DNA lesions and restarting stalled replication (Im et al., 2013). The recruitment of PrimPol to stalled forks seems to be via its direct interaction with the OB-C domain of RPA (Wan et al., 2013). The RPA/PrimPol interaction also allows repriming at the exposed ssDNA regions formed in the leading strand upon replisome stalling (Martinez-Jimenez et al., 2017). In addition, biochemical analysis has revealed that RPA stimulates the primase activity of PrimPol (Guilliam et al., 2017; Martinez-Jimenez et al., 2017).

Human DNA helicase B, known as HDHB, is another protein which interacts with RPA and is recruited to chromatin under RS-induced conditions (Guler et al., 2012). The RPA70/HDHB interaction promotes HDHB recruitment to chromatin following fork stalling induced by UV irradiation, camptothecin, or HU (Guler et al., 2012). RPA also modulates the activity of other two important DNA helicases, WRN and BLM. It has been shown that WRN can unwind DNA duplexes up to 850 nt in the presence of RPA, whereas WRN alone (without RPA) poorly processes DNA (Brosh et al., 1999). Qin et al. has identified that while high concentration of BLM can unwind dsDNA from a nick unidirectionally in the absence of RPA, the presence of RPA permits BLM’s unwinding in two opposite directions from a nick (Qin et al., 2020). These results suggest that RPA coating of the newly generated ssDNA can enhance helicase activities. RPA may also prevent ssDNA from annealing back to itself or forming secondary structures that may give rise to RS. The precise interplay between DNA helicases and RPA remains to be elucidated.

## BRCA2

### BRCA2 Protein Structure and Domains

BRCA2 is a tumor suppressor that plays a major role in DNA repair pathways and has been found recently in the protection of replication forks. It was discovered from breast cancer genome linkage studies in 1994 (Wooster et al., 1994), and it is well established that detrimental BRCA2 mutations are a major risk factor for breast and ovarian cancers (Antoniou et al., 2003). The human BRCA2 gene is located on chromosome 13q12.3 and contains 27 exons that translate into a protein of 3418 amino acids in length with molecular weight of approximately 390 kDa. A number of structural elements in BRCA2 have been identified, including eight BRC repeats which bind to monomeric RAD51 (Bork et al., 1996; Bignell et al., 1997; Wong et al., 1997), one helix-turn-helix (HTH) motif and three OB folds that together comprise a ssDNA-binding domain (Yang et al., 2002), and the C-terminal TR2 domain which stabilizes RAD51 nucleofilaments (Esashi et al., 2007; Figure 1A). Due to its large size, the structure of full-length BRCA2 structure was not available until 2014. Transmission electron microscopy analysis shows that BRCA2 exists as a homodimer (Shahid et al., 2014). BRCA2 predominantly resides in the nucleus with two nuclear localization signals flanking the TR2 domain (Yano et al., 2000) and one masked nuclear export signal in between the HTH motif and OB folds (Jeyasekharan et al., 2013; Figure 1A). BRCA2 acts as a platform to form multimeric structures–it not only directly binds to RAD51 but also to Partners with Localizer of BRCA2 (PALB2/FANCN) (Sy et al., 2009; Zhang et al., 2009) and Fanconi Anemia (FA) Complementation Group D2 (FANCD2) (Hussain et al., 2004). The role of BRCA1, PALB2, and BRCA2 as a complex in HR-mediated DSB repair has been well documented and are not be covered in this review. Instead, we focus on recent findings on the function of BRCA2 in replication fork processing.

### BRCA2-Mediated Recruitment of RAD51 to Stalled Forks

When replication fork is stalled, ssDNA generated at stalled forks is bound by RPA which is then replaced by RAD51. Phosphorylated RPA promotes binding to PALB2 to the stalled forks (Murphy et al., 2014). PALB2, which has been shown to colocalize with BRCA2 after RS in HeLa cells (Buisson et al., 2014), interacts with N-terminal domain of BRCA2, bringing BRCA2 to stalled forks (Hartford et al., 2016; Figure 3). Then BRCA2 recruits RAD51 to stalled forks by directly interacting with polo-like kinase 1 (PLK1) through its N-terminal CDK2-phosphorylated site (T77) and the polo box domain of PLK1 (Yata et al., 2014). Moreover, BRCA2-mediated RAD51 recruitment is assisted by FANCD2 and biorientation of chromosomes in cell division 1 like (BOD1L) (Hussain et al., 2004; Schlacher et al., 2012; Higgs et al., 2015; Table 1).

Recent studies show that the PDS5-wings apart-like protein homolog (WAPL) complex, a cohesin-associated factor that releases cohesin from chromosomes, is also involved in replication fork progression (Carvajal-Maldonado et al., 2019; Morales et al., 2020). Cohesin binds to chromatin in a multi-subunit complex that mediates cohesion between sister chromatids, but its role in replication and transcription remains unclear. PDS5 depletion leads to fork stalling in the absence of genotoxic stress and prevents the recruitment of WRN helicase-interacting protein 1 (WRNIP1), RAD51, and BRCA2 (Morales et al., 2020). The iPOND analysis has revealed that PDS5 is loaded onto replication forks regardless of BRCA2 presence (Carvajal-Maldonado et al., 2019). These results suggest that PDS5-WAPL complex is involved in the very early events of replication fork stalling.

### The Role of BRCA2 in Fork Protection

As mentioned above, fork remodeling/reversal is a key protective mechanism to stabilize stalled forks. However, reversed forks are prone to nucleolytic degradation by multiple nucleases including MRE11, EXO1 (exonuclease 1), CTIP (C-terminal binding protein interacting protein), and DNA2. Obviously, fork protection (FP) mechanisms are needed to antagonize nuclease degradation of reversed forks in order to preserve fork stability.

MRE11 is recruited to forks by many proteins, including mixed-lineage leukemia proteins 3 and 4 (MLL3/4), pax transactivation domain-interacting protein (PTIP), and chromodomain helicase DNA-binding protein 4 (CHD4) (Ray Chaudhuri et al., 2016), poly (ADP-ribose) polymerase 1 (PARP1) (Ding et al., 2016), RAD52 (Mijic et al., 2017), and sterile alpha motif domain and histidine-aspartic domain-containing protein 1 (SAMHD1) (Coquel et al., 2018). There are many studies indicating that BRCA2 is a key player in protecting forks from MRE11 degradation. After inducing RS by HU treatment in BRCA2-deficient cells, Y-shaped DNA intermediates as observed on two-dimensional gel electrophoresis disappear quickly, indicating uncontrolled degradation (Lomonosov et al., 2003). Examination of FP through DNA fiber assays (Schlacher et al., 2011; Ying et al., 2012) and electron microscopy analysis (Lemacon et al., 2017; Mijic et al., 2017) in BRCA2-depleted cells have shown that BRCA2 can protect nascent strand DNA from the degradative effect of MRE11 (Figure 3). Such protection appears to rely on cyclin-dependent kinase phosphorylation of BRCA2 at the serine 3291 position. BRCA2 S3291A mutant abrogates RAD51 from binding to the C-terminal TR2 domain of BRCA2 and thus prevents RAD51 nucleofilament formation (Esashi et al., 2005; Davies and Pellegrini, 2007). Interestingly, this mutant still has HR activity but abolishes FP (Schlacher et al., 2011; Feng and Jasin, 2017). Furthermore, expression of a BRC4 peptide, a BRC repeat from BRCA2 that disrupts RAD51 nucleofilaments, also promotes nascent strand degradation (Schlacher et al., 2011). BRCA2 does not need to interact with DNA in order to provide FP, suggesting that the crucial FP ability of BRCA2 is to recruit and stabilize RAD51 nucleofilament at stalled forks (Schlacher et al., 2011).

Besides MRE11, EXO1, and CTIP also degrade nascent strand and their depletion restores FP in BRCA1/2-deficient cells (Lemacon et al., 2017). In BRCA2-deficient cells, the role of DNA replication helicase/nuclease 2 (DNA2) is somewhat controversial since one group utilizing a small-molecule DNA2 inhibitor, C5, showed similar levels of rescue from strand degradation as with MRE11 inhibitor, mirin (Schlacher et al., 2011), while DNA2 depletion with siRNA did not provide FP (Lemacon et al., 2017). However, cells that are deficient in BOD1L (Higgs et al., 2015), RecQ1 helicase (Thangavel et al., 2015), or Abraxas brother 1 (ABRO1) (Xu et al., 2017) suffer from hyper-resection due to DNA2. U2OS cells under prolonged RS with HU treatment also have stalled forks that are degraded by DNA2 but not MRE11, EXO1, or CTIP (Thangavel et al., 2015). In addition, DNA2 and Werner syndrome ATP-dependent helicase (WRN) are involved in resection of ssDNA not protected by RAD51 (Wang et al., 2015), and both are implicated in replication fork restart (Thangavel et al., 2015).

MUS81 and SLX4 are endonucleases that are better known for resolving Holliday junctions during FA repair (Fekairi et al., 2009; Svendsen et al., 2009). However, MUS81 and SLX4 have also been shown to play a role at stalled replication forks (Hanada et al., 2007; Franchitto et al., 2008; Pepe and West, 2014; Lemacon et al., 2017; Porebski et al., 2019), and MUS81 promotes replication restart. In BRCA2-deficient cells, loss of MUS81 leads to increased levels of partially resected reversed forks with ssDNA tail and fewer DSBs (Lemacon et al., 2017). Conversely, MRE11 inhibition or EXO1 knockdown decreased both nascent strand degradation and formation of DSBs. These results suggest that MRE11 or EXO1 resection at reverse fork generates ssDNA substrate for MUS81 to cleave and then promotes fork restart at least in BRCA2-deficient cells (Lai et al., 2017; Lemacon et al., 2017). MUS81 is recruited to the chromatin during BRCA2 deficiency but not by loss of BRCA1 and is mediated by enhancer of zeste homolog 2 (EZH2), a histone-lysine-N-methyl transferase, through its methylation of histone H3 at lysine 27 at stalled forks (Rondinelli et al., 2017). On the other hand, SLX1-SLX4 endonucleolytic activity as well as DNA2 at stalled forks is inhibited by WRNIP1, and thus the FP provided by WRNIP1 is mechanistically distinct from BRCA2 (Porebski et al., 2019). Taken together, different subsets of nucleases are involved in nascent strand degradation at stalled replication forks, and different FP proteins are utilized to prevent these nucleases from working in an unregulated manner.

## CST

### Structure and Properties

The CST complex is a heterotrimeric protein composed of conserved telomere maintenance 1 (CTC1), suppressor of Cdc13 homolog (STN1), and TEN1 (Telomere Length Regulation Protein TEN1 Homolog). It is evolutionarily conserved from budding yeast (*Saccharomyces cerevisiae*) to human. In budding yeast it is known as Cdc13-Stn1-Ten1 complex, however fission yeast lacks the CTC1/Cdc13 homolog, but contains Stn1 and Ten1 (Martin et al., 2007). Recent cryo-EM structure of human CST reveals that it is capable of forming a decameric supercomplex when bound to telomeric ssDNA (Lim et al., 2020). CTC1 is the largest subunit with a molecular weight of 134 kDa, and it possesses seven OB-fold domains (Lim et al., 2020). STN1 is 44 kDa and TEN1 is 13.8 kDa with one OB-fold each (Figure 1A; Rice and Skordalakes, 2016). The CST complex is thought to resemble the RPA complex, in that STN1-TEN1 and RPA32-RPA14 share structural similarity and also have comparable domain organizations (Sun et al., 2009). The only difference is the presence of two winged-helix-turn-helix (wHTH) domains in STN1 but only one WH domain in RPA32 (Gelinas et al., 2009; Figure 1A). While RPA binds to ssDNA in a sequence independent manner, CST has a preference for G-rich sequences when substrates are short (Hom and Wuttke, 2017), but such preference decreases with the increase in length of the nucleotide (Miyake et al., 2009). The OB-fold of STN1 seems to play an important role in its preference for G-rich sequence, because mutation in the OB-fold of STN1 leads to decrease in binding to short G-rich sequences (Bhattacharjee et al., 2016; Hom and Wuttke, 2017). CST also binds to ss-dsDNA junctions in a sequence independent manner and needs shorter nucleotides for binding (Bhattacharjee et al., 2017). Although TEN1 is not required for DNA binding, it stabilizes the interaction of CTC1-STN1 (Feng et al., 2018). In addition, CST melts G4 structure and prevents its formation, thus facilitating replication of telomeric DNA and other G-rich regions (Bhattacharjee et al., 2017; Zhang et al., 2019). The OB folds of CST also play an important role in protein-protein interactions (Ganduri and Lue, 2017; Shastrula et al., 2018). Budding yeast Cdc13 consists of four OB-folds which function in ssDNA binding, Cdc13 homo-dimerization, protein-protein interaction, and DNA polymerase α-primase binding (Hughes et al., 2000; Sun et al., 2011; Lewis et al., 2014).

Unlike RPA and BRCA2, CST is a relatively new member in genome maintenance. Knockdown of CTC1 or STN1 elevates the level of multi-telomeric signals and telomere instabilities (Surovtseva et al., 2009; Dai et al., 2010) and increases the formation of anaphase bridges, micronuclei, chromosome breakage, and chromosome pulverization (Stewart et al., 2012; Chastain et al., 2016; Lyu et al., 2019b). Disease-causing CTC1 mutations induce spontaneous chromosome instabilities that are further increased by RS (Wang and Chai, 2018). In budding yeast, Cdc13 deficiency also leads to genome stability in the form of unstable chromosomes (Langston et al., 2020). Recent studies have shown that CST plays a multifaceted role in genome maintenance. Here, we review the well-studied role of CST in telomere maintenance, followed by describing its functions in genome stability maintenance at non-telomeric regions.

### Role of CST in Telomere Maintenance

In budding yeasts, CST binds to the single-stranded region at telomeres, plays an essential role in telomere protection, and also functions in telomere replication by recruiting telomerase. The telomere elongation and protection function of yeast CST is tightly regulated by phosphorylation events which occur in a cell cycle-dependent manner. The telomere protection function of Cdc13 occurs through its interaction with Stn1 and Ten1, forming a stable CST complex, which is mediated by Cdk1-dependent phosphorylation of Stn1 and SUMOylation of Cdc13 (Hang et al., 2011; Liu et al., 2014). For telomere elongation, Cdc13 is recruited to the 3′ telomeric end which is mediated by its interaction with an accessory subunit of the yeast telomerase complex Est1 through its recruitment domain. This interaction is favored by both the increased abundance of the two proteins and also phosphorylation of Cdc13 by Cdk1, Mec1 and Tel1 which occurs in the late S phase to G2 phase of the cell cycle (Evans and Lundblad, 1999; Tseng et al., 2006; Li et al., 2009; Wu and Zakian, 2011). During G2/M phase the interaction is disrupted by other dephosphorylation and phosphorylation of Cdc13 by phosphatase 2A (PP2A) subunit Pph22 and the yeast Aurora kinase homolog Ipl1, respectively (Shen et al., 2014).

**FIGURE 4:**
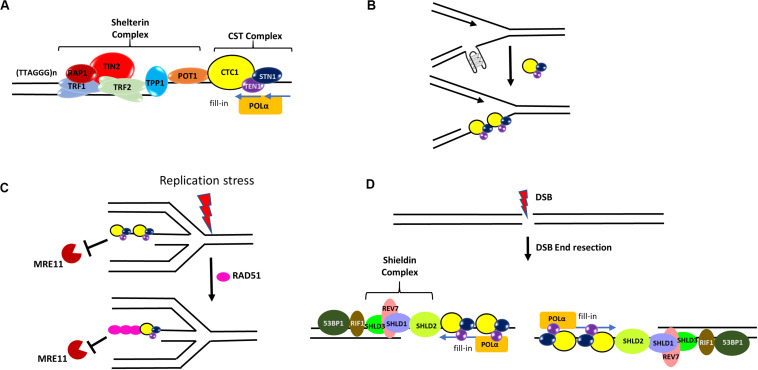
Roles of CST in genome maintenance. **(A)** CST at telomeres. In human cells, CST complex interacts with the TPP1-POT1 subunit of shelterin and promotes efficient replication of telomeres. It also stimulates the C-strand fill-in activity of DNA polymerase α-primase (POLα). **(B)** At elongating replication forks, CST may resolve or prevent the formation of G4s that hinder DNA replication. **(C)** During stalled forks, CST protects the reversed fork against MRE11 degradation by directly blocking MRE11 access to reversed forks and also facilitating the recruitment of RAD51 to forks. **(D)** During DSB repair, the Shieldin complex (SHLD1-SHLD2-SHLD3-REV7) localizes to DSB sites in a 53BP1- and RIF1-dependent manner. It has been hypothesized that CST may recruit POLα to DSB ends to fill in resected DSB ends.

In humans, the main telomere maintenance complex is Shelterin (a six subunit complex consisting of TRF1, TRF2, TIN2, RAP1, POT1, TPP1) which binds to both the double-stranded and single-stranded telomeric region (Giraud-Panis et al., 2010; Price et al., 2010; Gu et al., 2012; Rice and Skordalakes, 2016). While human CST complex does not function in telomere capping, it is important for the synthesis of the lagging strand telomeres and also mediates C-strand fill-in through its interaction with TPP1-POT1 and with the help of DNA polymerase α-primase (POLα) (Huang et al., 2012; Lue et al., 2014; Feng et al., 2018; Table 1 and Figure 4A), thus helping in the formation of t-loop. In fact, CTC1 and STN1 was initially identified as POLα accessory factor (AAF) AAF132 and AAF44, respectively, as they stimulate the primase and DNA synthesis activities of POLα (Goulian and Heard, 1990; Casteel et al., 2009). Interaction of STN1 with the POLA2 subunit of POLα is important for such stimulation (Ganduri and Lue, 2017). Depletion of CTC1 or STN1 results in lengthened G-overhangs as the C-strand fill-in becomes defective (Surovtseva et al., 2009; Dai et al., 2010; Huang et al., 2012). TEN1 is also essential for C-strand synthesis and TEN1^–/–^ cells exhibit progressive telomere shortening (Huang et al., 2012; Kasbek et al., 2013; Feng et al., 2018).

In addition to C-strand fill-in, CST facilitates telomeric DNA replication. STN1 depletion reduces the rate of replication of the telomeric duplex region (Stewart et al., 2012). It has been shown that CST promotes efficient restart of stalled replication at telomeres by helping RAD51 load onto telomeres (Chastain et al., 2016). CST also helps in restricting of telomerase activity through primer sequestration and physical interaction with POT1–TPP1, which is the telomerase processivity factor (Chen et al., 2012). Both CTC1 and STN1 are required whereas TEN1 is dispensable for this activity (Feng et al., 2018).

### Functions of CST in Protecting Global Genome Stability Under RS

Only ∼20% of STN1 foci localize at telomeres (Miyake et al., 2009), and CTC1 and STN1 were originally identified as a POLα stimulatory factor (Goulian and Heard, 1990; Casteel et al., 2009). These early observations provide the initial evidence that CST possesses functions outside telomeres in particular in global DNA replication progression (Derboven et al., 2014; Wang Y. et al., 2019). CST is capable of preventing the accumulation of G4 structures during unperturbed DNA replication (Bhattacharjee et al., 2017; Figure 4B), and STN1 depletion increases G4 formation and slows bulk genomic DNA replication (Zhang et al., 2019). Recently, CST’s role in active replication is reported to be in regulating origin licensing through its interaction with the MCM complex and disrupting the binding of CDT1 to MCM (Table 1). CST also enhances replisome assembly by promoting AND-1/POLα chromatin association (Wang Y. et al., 2019; Table 1).

Several lines of evidence demonstrate that CST plays a prominent role at stalled replication forks. First, CST over-expression increases replication recovery from HU- and aphidicolin-induced fork stalling (Wang et al., 2014). Second, CST is needed in stoichiometric amounts to facilitate re-initiation of DNA replication at repaired forks and/or dormant origins. CST increases the firing of late or dormant origins following release from HU treatment (Wang et al., 2014). Third, we have shown that CST is important for maintaining the stability of GC-rich repetitive sequences genome-wide under HU induced RS. STN1 is enriched at GC-rich repetitive sequences after HU treatment. Fluorescence *in situ* hybridization (FISH) analysis reveals that these STN1-binding sites are prone to breakage and cause chromosome fragmentation in STN1 deficient cells (Chastain et al., 2016). HU or APH treatment induces CST interaction with RAD51 in an ATR-dependent manner (Chastain et al., 2016; Table 1). Suppression of each CST subunit impairs HU-induced RAD51 foci formation as well as RAD51 binding to GC-rich repetitive sites, suggesting that CST may facilitate the recruitment of RAD51 to stalled sites after HU-induced RS (Chastain et al., 2016; Figure 4C). CST is also recently shown to be localized at stalled replication fork and stabilize the fork by blocking MRE11-mediated nascent strand degradation (Lyu et al., 2019b; Figure 4C). These findings provide a mechanistic link between CST and other key players in fork stabilization and fork restart, at least at G-rich sequences. Since the stable G4 structure poses a special challenge to replication machinery, it will be interesting to determine how CST regulates RAD51 activity at G4-forming stalled sites, including whether it promotes RAD51 filament formation or strand invasion activity at these sites.

Recently, the role of CST in DSB repair via canonical non-homologous end joining is reported, where CST interacts with the Shieldin complex (SHLD1-SHLD2-SHLD3-REV7) and counteracts DSB end resection in a 53BP1–RIF1–Shieldin dependent manner in BRCA1 mutated cells (Barazas et al., 2018; Dev et al., 2018; Mirman et al., 2018; Table 1). It has been proposed that CST may recruit POLα to DSB ends to fill in resected DSB ends (Mirman et al., 2018)–an intriguing hypothesis that remains to be tested (Figure 4D). Nonetheless, while the role of CST in telomere maintenance itself is important for the genome stability, the emerging non-telomeric functions of CST enhance its importance in maintaining global genome stability.

### CST and Disease

Two important diseases associated with mutations in CST are Coats plus syndrome (CP) and dyskeratosis congenita (DC). Coat plus is an autosomal recessive disorder where patients show intrauterine growth retardation, intracranial calcifications, retinopathy, and gastrointestinal bleeding (Anderson et al., 2012; Simon et al., 2016). DC is another rare genetic disorder characterized by lacy reticular pigmentation of the upper chest and/or neck, oral leukoplakia, and bone marrow failure (Nelson and Bertuch, 2012). Characterization of pathogenic CTC1 and STN1 mutations shows diverse molecular defects affecting the telomeric and as well as non-telomeric function of CST. This includes inability to form the CST complex, accumulation of internal single-stranded gaps of telomeric DNA, defect in interaction with POLα, telomere DNA replication defects, deficiency in interaction with RAD51, increase in spontaneous γ H2AX staining, chromosome breakage and fragmentation causing global genome instability (Dai et al., 2010; Chen et al., 2013; Gu and Chang, 2013; Wang and Chai, 2018). Further investigation will be helpful to dissect the roles of various molecular features of CST in disease pathogenesis.

## Relationship Between CST, RPA, and BRCA2

As described above, CST shares structural similarities with RPA and was initially thought to be a telomeric alternative of RPA for protecting the integrity of special telomeric sequence and structure. The discovery of its non-telomeric function in global RS response has prompted great interests in understanding the spatial and temporal relationships between RPA and CST during RS response. RPA is abundant and binds to ssDNA with high affinity. In contrast, CST is difficult to detect in cells. The low abundance of CST may partially explain why iPOND has not been successful in detecting CST at stalled forks. Using the SIRF (*in situ* protein interactions at nascent and stalled replication forks) assay, we are able to detect CST at stalled forks (Lyu et al., 2019b), thus providing direct evidence that CST also localizes at stalled forks. Many questions remain to be answered in order to fully understand the genome maintenance mechanisms in response to fork stalling. Do RPA and CST bind to the same ssDNA formed at stalled forks or do they localize at different stalled sites? Do they compete for binding to ssDNA? Does CST binding to DNA also play a role in ATR signaling like RPA? Does CST interact with a set of proteins distinct from RPA-interacting proteins and modulate the activities of these proteins?

Likewise, BRCA2 and CST share a few striking functional similarities. Both proteins interact with RAD51, promote the recruitment of RAD51 to stalled forks, and protect reversed forks from unscheduled MRE11 degradation of nascent strand DNA. It will be important to know whether BRCA2 and CST protect fork stability in the same pathway or in parallel pathways. If they are in parallel pathways, do they protect forks stalled at different regions in the genome? While it is tempting to speculate that CST may be a RAD51 mediator by displacing RPA from ssDNA in a manner similar to BRCA2-DSS1, it has been reported that the DNA-binding ability of BRCA2 is dispensable for FP (Schlacher et al., 2011) while CST binding to DNA is required for FP (Lyu et al., 2019b), suggesting that there may be a fundamental difference underlying FP mechanisms by BRCA2 and CST. In addition, CST differs from BRCA2 in that it mediates POLα fill-in synthesis at telomere ends (Dai et al., 2010; Huang et al., 2012), and it has been proposed that CST/POLα-dependent fill-in synthesis may counteract end resection at DSB ends (Mirman et al., 2018). It remains to be determined whether such fill-in synthesis plays a significant role in countering nucleolytic degradation of nascent strand DNA at reversed forks. Understanding the relationship and interplay between RPA, BRCA2, and CST will provide novel insights into the genome protection mechanism.

## Novel Cancer Diagnostic and Therapeutic Approaches

The intrinsic level of RS in cancer cells is notably elevated compared to normal cells as a result of rapid proliferation, aberrant origin firing due to oncogene expression, loss of cell cycle checkpoint activation, and/or deficiency in repairing DNA damage. Such elevated RS level can be exploited in cancer therapy through further increase of RS, which then produces high levels of genome instability that lead to cancer cell death. Many traditional chemotherapeutic drugs such as alkylating agents (including cyclophosphamide, melphalan, temozolomide, etc.) and platinum-containing agents (including carboplatin, cisplatin, and oxaliplatin) produce DNA damage and severely perturb DNA replication. Their therapeutic effects can be attributed in part to their abilities to induce high levels of RS (Dobbelstein and Sorensen, 2015). In particular, tumors that are deficient in repairing DNA damage caused by RS are particularly vulnerable to these drugs. Given the important roles of OB-fold proteins such as RPA, BRCA2, and CST in RS pathways, targeting these proteins and their interacting partners may be a promising novel therapeutic approach in combination with traditional therapies.

RPA is the first responder in the ATR pathway, thus blocking the function of RPA is believed to be a promising strategy for cancer treatment. Downregulation of RPA14 has been shown to inhibit human gastric adenocarcinoma growth in a xenograft model (Dai et al., 2017). While there has been no FDA-approved anticancer drugs that target RPA, a recent high throughput screening of 2,000 small molecules has identified 9 potential candidates for inhibition of RPA binding activity after two rounds of screening (Andrews and Turchi, 2004). One of them has shown *in vivo* efficacy in models of epithelial ovarian cancer (EOC) and non-small cell lung cancer (NSCLC) (Mishra et al., 2015). The same group has also developed a series of novel compound analogs with low micromolar RPA inhibitory activity, increased solubility, and easier cellular uptake (Gavande et al., 2020). However, RPA binding to ssDNA is a crucial initiator of both HR-mediated repair and resolution of RS, and directly affecting its function could promote genomic instability. Since RPA has many binding partners (Table 1), targeting its binding partners may offer better therapeutic strategies to circumvent this hurdle.

BRCA2 along with BRCA1 are well-known tumor suppressors and thus typically deleted or functionally deficient in tumors. PARP1 inhibitors (PARPi) have been developed against BRCA1/2-deficient tumors and are quite effective. With deficient HR-mediated DSB repair due to the missing BRCA1/2, PARP inhibition increases single-strand breaks and traps PARP on DNA, leading to blocked replication and eventually apoptosis (Lord and Ashworth, 2017). Unfortunately, tumors have acquired resistance to PARPi partially due to the restoration of HR (Lord and Ashworth, 2017) and/or rescue of replication fork stability (Chaudhuri et al., 2016; Michl et al., 2016). In this regard, targeting of POLθ, which is involved in microhomology-mediated end joining (Mateos-Gomez et al., 2015), RAD52, which becomes an important HR factor in these cells (Feng et al., 2011; Lok et al., 2013), and FANCD2, that is overexpressed to overcome RS (Michl et al., 2016), are all viable strategies for treating PARPi-resistant BRCA1/2-deficient tumors. In addition, inhibition of nucleases in fork degradation such as MUS81 and SLX4 also promotes apoptosis in BRCA2-deficient tumors (Minocherhomji et al., 2015; Lai et al., 2017) and may enhance patient survivability.

The emerging role of CST in maintaining genome stability suggests that CST could be a good therapeutic target. STN1 suppression has been shown to enhance the cytotoxic effect of several chemotherapeutic agents (Lee et al., 2016). Loss of SHLD1/SHLD2 which interacts with CST and counteracts DSB end resection is shown to confer hypersensitivity to the DNA-crosslinking agent cisplatin (Dev et al., 2018). In a human melanoma cell model, downregulation of CTC1 enhances the radiosensitivity by inducing DNA damage and promoting telomere shortening, thus making it an attractive target for the treatment of human melanoma (Luo et al., 2014). CST also plays a role in telomere maintenance in ALT cells (Huang et al., 2017), therefore suppressing CST may be a potential therapeutic approach for inhibiting the growth of ALT-positive cancer cells. Despite its important function in telomere maintenance and non-telomeric function in genome stability, no chemotherapeutics drugs have been developed to target the CST complex yet. This may be in part due to the difficulty in solving the structure of CTC1, although STN1-TEN1 structure was solved several years ago (Bryan et al., 2013). The recent availability of the cryo-EM structure of the whole CST complex is expected to facilitate this process (Lim et al., 2020).

## Conclusion

OB-fold proteins, covered above, are some of the major players in maintaining genome stability. While significant progress has been made, especially with RPA and BRCA2, others such as CST require more in-depth studies to understand not only their functions but also interactions with other protein complexes and broader cellular physiological interplay between mitosis, replication, repair, apoptosis, and their regulations in both normal tissues and tumors. This need for better understanding especially in the context of current combinatorial cancer therapy is highlighted by the fact that BRCA2-deficient tumors under dual PARP inhibition and MUS81 depletion have improved viability compared to either alone (Rondinelli et al., 2017). Future studies that enhance our understanding of interaction between these proteins will produce novel therapeutic modalities in combination with current agents for the treatment of cancers.

## Author Contributions

All authors drafted the manuscript, contributed to manuscript revision, and approved the submitted version.

## Conflict of Interest

The authors declare that the research was conducted in the absence of any commercial or financial relationships that could be construed as a potential conflict of interest.
